# Blazed Gratings Recorded in Absorbent Photopolymers

**DOI:** 10.3390/ma9030195

**Published:** 2016-03-15

**Authors:** Roberto Fernández, Sergi Gallego, Andrés Márquez, Víctor Navarro-Fuster, Augusto Beléndez

**Affiliations:** 1 Instituto Universitario de Física Aplicada a las Ciencias y las Tecnologías, Universidad de Alicante, Apartado 99, E03080 Alicante, Spain; roberto.fernandez@ua.es (R.F.); andres.marquez@ua.es (A.M.); vinafu@gmail.com (V.N.-F.); a.belendez@ua.es (A.B.); 2 Departamento de Física, Ingeniería de Sistemas y Teoría de la Señal. Universidad de Alicante, Apartado 99, E03080 Alicante, Spain; 3 Departamento de Óptica, Farmacología y Anatomía. Universidad de Alicante. Apartado 99, E03080 Alicante, Spain

**Keywords:** photopolymers, optical recording materials, diffractive elements

## Abstract

Phase diffractive optical elements, which have many interesting applications, are usually fabricated using a photoresist. In this paper, they were made using a hybrid optic-digital system and a photopolymer as recording medium. We analyzed the characteristics of the input and recording light and then simulated the generation of blazed gratings with different spatial periods in different types of photopolymers using a diffusion model. Finally, we analyzed the output and diffraction efficiencies of the 0 and 1st order so as to compare the simulated values with those measured experimentally. We evaluated the effects of index matching in a standard PVA/AA photopolymer, and in a variation of Biophotopol, a more biocompatible photopolymer. Diffraction efficiencies near 70%, for a wavelength of 633 nm, were achieved for periods longer than 300 µm in this kind of materials.

## 1. Introduction

Photopolymers are frequently used in many optical applications such as security holograms or holographic memories [1,2,3], astronomical applications [4] or spectroscopy [5] because of their versatility, low cost and chemical composition that permits new components to be introduced to modify their properties.

One of these applications is the fabrication of diffractive optical elements (DOEs) based on two properties: the relief structures formed on the surface due to variations in thickness, and the refractive index modulation distribution [6,7]. These properties have been analyzed in detail, together with the effects of using different chemical compositions, index matching substances and covering architectures [8,9,10].

One of the photopolymers most widely studied is poly (vinyl)alcohol acrylamide (PVA/AA) [9,10,11,12]. In this material, as in other photopolymers, a grating is formed due to photopolymerization and mass transport processes. 

The undesirable feature of these AA based photopolymers is the high toxicity of their components, in particular, of the main monomer AA, and their low environmental compatibility. Some efforts have been made to replace this component in the chemical formulation so as to design highly environmentally compatible photopolymers [13,14,15,16]. One of the greenest photopolymers is called Biophotopol [16], which uses sodium acrylate (NaAO) as the main monomer. A variation of this material, where we introduced a crosslinker monomer and use the same dye of PVA/AA, is also analyzed in this paper. The results obtained by this more environmentally compatible photopolymer, PVA/NaAO, are compared with those obtained using the PVA/AA.

A wide range of values of monomer diffusion in these materials have been reported in the literature, e.g. 10^−7^ to 10^−11^ cm^2^/s [9,10,11,12]. One effect of values greater than 10^−7^ cm^2^/s is that changes in the surface relief profile due to mass transfer make it difficult to produce sharp structures such as blazed gratings on the layer. To improve the recording of this kind of structure, a paraffin (*n* = 1.478) can be used to seal the materials to a cover plate. This results in a significant reduction in the estimated monomer diffusion and a consequent improvement in the sharpness of the recorded diffraction optical element (DOEs) [17]. A blazed grating can theoretically reach diffraction efficiency (DE) of 100% and is an important DOE due to its potential applications in optical communications. Such gratings are more complex than the sinusoidal gratings usually recorded in this kind of recording material and it is interesting to study the capability of the diffusion model to reproduce the behavior of the material and compare it with experimental measurements.

We used a Liquid Crystal on Silicon (LCoS) spatial light modulator (SLM) to obtain the desired amplitude recording intensity distribution projected on the material. Our aim was to obtain a linear response for each level of gray with good contrast and we characterized LCoS using the model proposed in [18]. As opposed to the lithographic technique, spatial light modulation provides a dynamic experimental setup and allows the desired DOE to be modified in real time. This is described in detail in the experimental section.

Another important aspect to take into account is the low pass filtering produced in the final DOE mainly due to the diaphragm placed in the focal plane of the relay lens to eliminate the diffraction orders produced by pixilation of the LCD. 

We compared the predictions using an accurate version of the model proposed in [17] that takes into account both the volume refractive index variations and thickness variations together with aspects like the effect of the visibility achieved with the LCD and the high frequency cut-off due to the diaphragm [19]. These results were compared with the experimental ones analyzing the diffraction efficiencies [20] of the main orders recorded on the material.

## 2. Theoretical Diffusion Model

As explained above, we used a coverplated, index matched photopolymer to prevent the transmitted light from being affected by variations in thickness. The following general equations were used to simulate the material behavior during polymerization [12]:

(1) $$\frac{\partial \left(M\right)\left(x,z,t\right)}{\partial t}=\frac{\partial }{\partial x}D_{m}\left(t\right)\frac{\partial \left(M\right)\left(x,z,t\right)}{\partial x}+\frac{\partial }{\partial z}D_{m}\left(t\right)\frac{\partial \left(M\right)\left(x,z,t\right)}{\partial z}-F_{R}\left(x,z,t\right)\left[M\right]\left(x,z,t\right)$$

(2) $$\frac{\partial \left(P\right)\left(x,z,t\right)}{\partial t}=F_{R}\left(x,z,t\right)\left[M\right]\left(x,z,t\right)$$

where *D_m_* is the monomer diffusion inside the material which decreases with time, *F_R_* is the polymerization rate and (*M*) and (*P*) are the volume fraction of the monomer and polymer, respectively. 

The polymerization rate depends on the reaction kinetics and the recording intensity; this dependence can be described by the following equation:

(3) $$F_{R}(x,y,z,t)=k_{R}(x,y,z,t)I{\left(x,y,z,t\right)}^{\gamma }=k_{R}(x,y,z,t)I{\left(x,y\right)}^{\gamma }e^{-\alpha (t)\gamma z}$$

where *I* is the recording intensity, *k_R_* is the polymerization constant, *γ* indicates the relationship between intensity and polymerization rate and *α* is the coefficient of light attenuation. The initial value of *α* [*α* (t = 0) *= α_0_*] can be obtained if the transmittance and the physical thickness of the layer are known. 

We assume that, during recording, the polymerization rate increases very quickly due to the Trommsdorff effect and then decreases because it is limited by the viscosity of the material [21]; thus, *k_R_* can be written as follows:

(4) $$k_{R}=k_{R_{0}}e^{-\alpha_{T}t}$$

where *α_T_* is the attenuation due to the Trommsdorff effect and *k_R0_* is the initial value of the polymerization rate.

There are different methods for solving these differential equations. In this paper we used the finite-difference method (FDM), a numerical method, to solve a three-dimensional problem. Therefore, Equation (1) and Equation (2) can be written as:

(5) $$M_{i,j,k}=M_{i,j,k}=\frac{\Delta t}{\Delta x^{2}}D_{m}M_{i+1,j,k-1}-2\frac{\Delta t}{\Delta x^{2}}D_{m}\left(t\right)M_{i,j,k-1}+\frac{\Delta t}{\Delta x^{2}}D_{m}\left(t\right)M_{i-1,j,k-1}+\;\frac{\Delta t}{\Delta z^{2}}D_{m}\left(t\right)M_{i,j+1,k-1}-\frac{2\Delta t}{\Delta z^{2}}D_{m}\left(t\right)M_{i,j,k-1}+\frac{\Delta t}{\Delta z^{2}}D_{m}\left(t\right)M_{i,j-1,k-1}-\Delta tF_{R_{i,j,k-1}}M_{i,j,k.1}+M_{i,j,k-1}$$

(6) $$P_{i,j,k}=P_{i,j,k}+\Delta t*F_{R_{i,j,k-1}}*M_{i,j,k-1}$$

In order to guarantee the numerical stability of the equations, the increment in the time domain, Δ*t*, must satisfy the stability criterion:

(7) $$\Delta t\leq \frac{{\left(\Delta x\right)}^{2}}{D_{m}}$$

In this case, we take Δ*t* = 0.1 (Δ*x*^2^/*D_m_*). 

Once we know the monomer and polymer concentrations, the refractive index values can be used to determine the refractive index distribution during the recording process. The refractive index distribution can be measured using the Lorentz–Lorenz equation as follows:

(8) $$\frac{n^{2}-1}{n^{2}+2}=\frac{{n_{m}}^{2}-1}{{n_{m}}^{2}+2}\left[M\right]+\frac{{n_{p}}^{2}-1}{{n_{p}}^{2}+2}\left[M,P\right]+\frac{{n_{b}}^{2}-1}{{n_{b}}^{2}+2}\left(1-M_{0}\right)$$

where *M_0_* is the average initial value of the volume fraction of monomer, *n_p_* is the polymer refractive index, *n_m_* is the monomer refractive index, and *n_b_* is the binder refractive index. The last two parameters can be measured using a refractometer and the value of *n_p_* can be obtained using the zero spatial frequency technique [22]. 

The light intensity distribution during the recording process takes a blazed form onto the material and can be written as follows:

(9) $$I=\frac{I_{0}}{\frac{1}{f_{s}∙\Delta_{x}}+1}$$

where *f_s_* is the period of the grating and I_0_ is the recording intensity, 0.5 mW/cm^2^.

## 3. Experimental Setup

A PVA/acrylamide (PVA/AA) based photopolymer and PVA/NaAO based photopolymer, both with a thickness of about 90 ± 5 µm measured by ultrasounds, for 30 mL solution deposited onto a square glass substrate 20 cm × 20 cm, were used to carry out the experiments. N-N’dimethyl-bis-acrylamide (BMA) was used as crosslinker to prevent polymer diffusion from illuminated to non-illuminated zones, improve the polymerization rate and increase the value of *n_p_* [16]. Triethanolamine (TEA) was used as co-initiator and yellowish eosin (YE) as dye. A small proportion of water was also added as additional plasticizer. The specific concentrations used in this study are shown in Table 1 and Table 2.

The composition was deposited on a glass substrate (20 cm × 25 cm) using the force of gravity and left in the dark (*Relative Humidity* = 40%–45%, *T* = 20–23 °C). Once most of the water had evaporated (after about 24 h), the layer had enough mechanical resistance to be cut without any deformation. 

In order to improve the recording of sharp elements and evaluate the effects of index matching, we used a paraffin with a refractive index *n* = 1.478, very similar to the average refractive index of the photopolymer (*n* = 1.477). 

**Figure 1:**
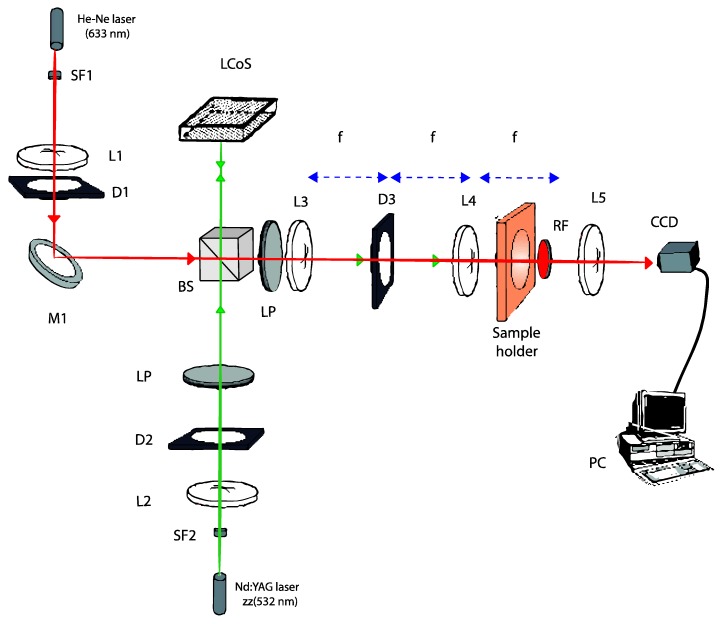
Experimental setup used to register and analyze in real-time the DOEs (blazed gratings): D, diaphragm; L, lens; BS, beam splitter; SF, spatial filter; LP, lineal polarizer; RF, red filter; M, mirror.

To evaluate the recording of sharp DOEs, the setup shown in Figure 1 was used. A solid-state Verdi laser (Nd-YVO4) with a wavelength of 532 nm (green light), at which the material exhibits maximum absorption, was used during the recording process. 

In the setup, we can distinguish two beams, the recording beam and the analyzing beam. The periodic pattern, in this case the blazed grating, is introduced by a Liquid Crystal on Silicon (LCoS) modulator placed along the recording arm of our setup and sandwiched between two polarizers (P) oriented to produce amplitude-mostly modulation. Then, with a 4f system the intensity distribution generated by the LCoS is imaged onto the recording material. In this work, we have used a recording intensity of 0.25 mW/cm^2^, because the different photopolymers tested present an acceptable response and we can analyze the diffraction efficiencies in real time. 

The analyzing arm is made up of a He-Ne laser at a wavelength of 633 nm, at which the material exhibits no absorption, used to analyze in real time the elements formed on the material. This arm is designed to collimate the light incident on the recording material and a diaphragm (D1) was used to limit the aperture of this collimated beam of light.

A non-polarizing beam splitter (BS) was used to make the two beams follow the same path up to the red filter (RF) placed behind the recording material to ensure that only the analyzing beam is incident on the CCD placed at the end of the setup. To separate the different diffraction orders, we placed a lens behind the material, obtaining the Fraunhofer diffraction pattern on the camera. We used a high dynamic range CCD camera model pco.1600 from pco.imaging. This camera has a resolution of 1600 × 1200 and a pixel size of 7.4 μm × 7.4 μm The camera was also used in the plane of the recording material to evaluate the intensity pattern actually imaged from the LCD plane.

**Figure 2:**
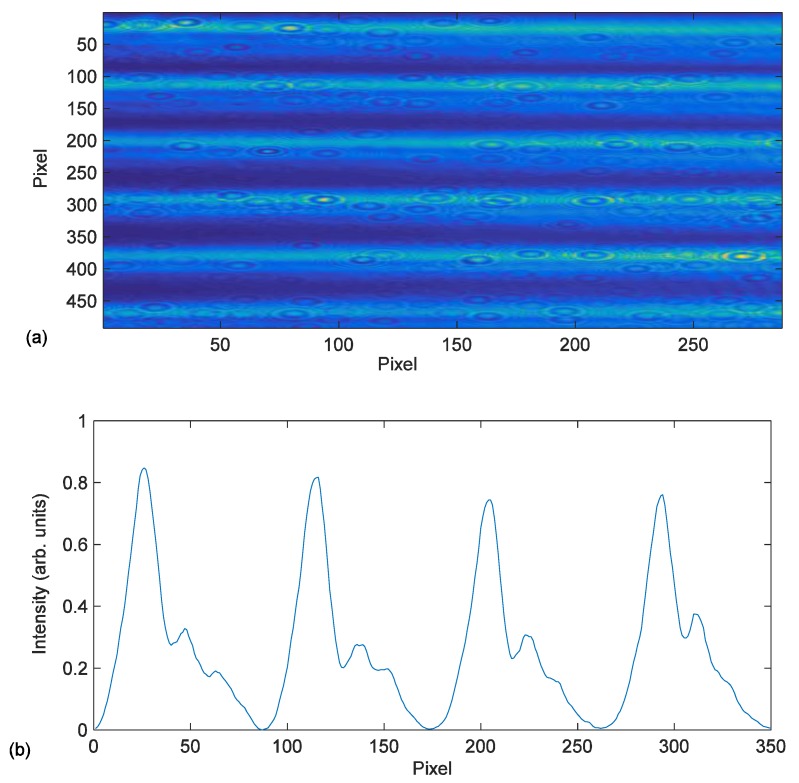
(**a**) The image on the photopolymer provided by the LCoS and captured by the CCD camera; and (**b**) the intensity profile provided by the LCoS across a vertical line of the image in (**a**).

The image obtained by the CCD camera located in the recording plane is shown in Figure 2a. and the intensity distribution of this image in Figure 2b. The Figure 2b shows the effects of low pass filtering on the final DOE due to the diaphragm used to eliminate the diffraction orders produced by pixilation of the LCD, as mentioned previously. This figure shows a smoothed form of the abrupt edges that take place on the recording intensity distribution with a grating period of 672 µm. We have introduced in the diffusion model the recording intensity captured by the CCD camera, Figure 2b, to be more accurate. 

This intensity pattern is the one that will be recorded on the photopolymeric material converted into a phase element. To simulate reality, this profile was introduced in the input of the diffusion model so that the output would take into account the filtering introduced by the system. As mentioned above, in order to improve the recording pattern of this type of sharp elements we used an LCoS with a pixel size of 8 µm as opposed to the transmission LCD with a pixel size of 44 µm used in previous studies. This new spatial light modulation opens up a great number of possibilities such as recording symmetric and asymmetric holographic patterns using a single beam [23] or cylindrical or spherical diffractive lenses [24] as well as greatly improving the resolution of diffractive optical elements, as in the case of our study. 

**Figure 3:**
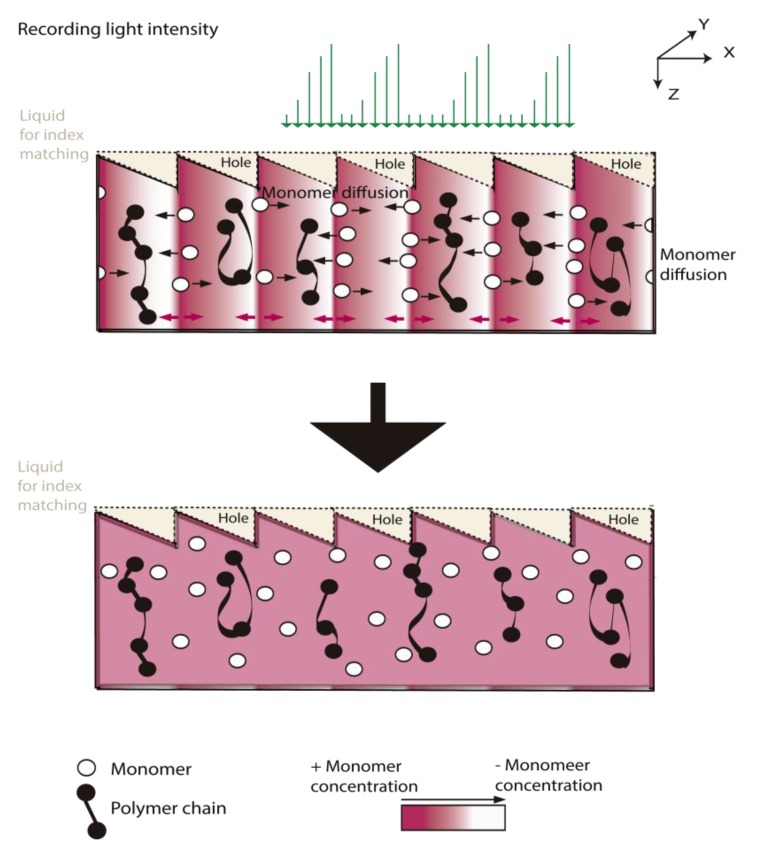
Diagram of the blazed grating recording in the photopolymers with index matching. The “apparent” diffusion is due to the recovering surface changes and the “real” diffusion to the internal monomer motion.

The recording process is represented in Figure 3. This figure firstly shows the shrinkage in the illuminated zones, mainly due to polymerization, making the material more compact. Secondly, after illumination, the illuminated zones swell due to monomer diffusion. The incorporation of a cover plate and index matching systems improves the conservation and lifetime of the recorded DOEs and prevents thickness variations. In addition, the reduction in mass transfer produced by the sealant [8] makes it possible to record sharp diffractive optical elements with insignificant smoothing of the refractive index profiles, as we show in this paper. In the introduction, we mentioned the relevance of blazed gratings due to their sharp profile and multiple applications that makes them very useful for characterizing the optical system and photopolymeric materials.

## 4. Results and Discussion

After controlling the beam shape projected onto the photopolymer, our aim was to analyze the influence of the material properties on the DOE recording. First we simulated blazed gratings with our model in order to analyze the influence of index matching and present the results for different spatial periods. Then, with a theoretical idea of the results and the influence of the spatial period and index matching, we compared these theoretical results with the experimental ones.

### 4.1. Simulation of the Recording of Blazed Gratings on PVA-AA Photopolymers

**Figure 4:**
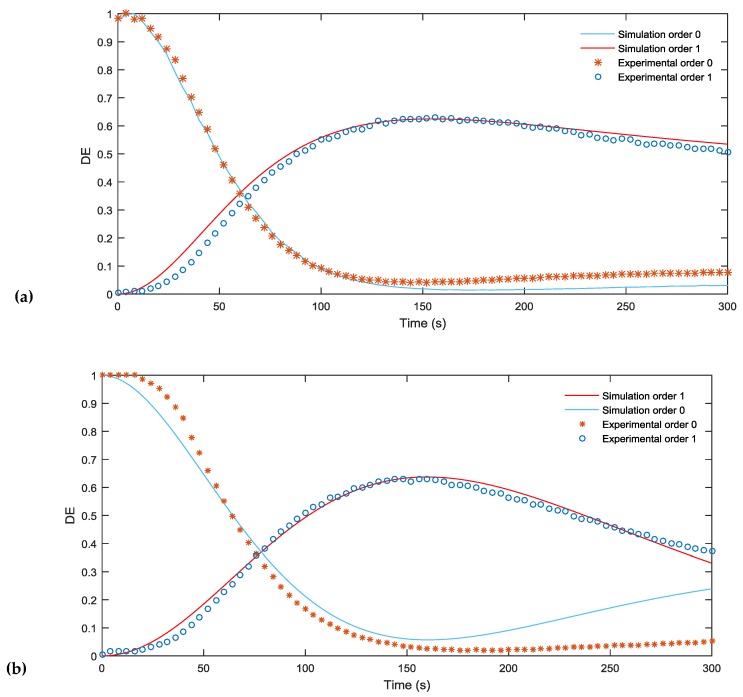
(**a**) Comparison of the simulated and experimental DE of a 672 µm blazed grating during an exposure time of 300 s; (**b**) Comparison of the simulated and experimental DE of a 336 µm blazed grating during an exposure time of 300 s.

In this section, we analyze the response of the material and the capability of our model to predict the material behavior taking into account the index matching. First, in Figure 4a,b, the simulated and experimental DEs are compared for blazed gratings with a period of 672 µm and 336 µm, respectively. It may be seen that in both figures the diffraction efficiency (DE) of the first order increases and reaches a maximum value of almost 70% after an exposure time of 150 s. This is a good result limited due to the low pass filtering produced in the experimental setup by the diaphragms, which eliminate pixilation of the grating before recording.

First of all, it can be seen that the validity of the model to represent the behavior of the material when recording blazed gratings of different periods is remarkable, as is the agreement between the results of the simulation and experimental measurements. The similarity in behavior at the two spatial periods means that there is very little monomer diffusion at these spatial frequencies.

Secondly, the DE decreases as the grating period is decreased since at shorter spatial periods the influence of low pass filtering and diffusion is greater. The phase shift, related to *Δn × d*, where *Δn* is the refraction index variation between dark zones and the zones where the intensity is maximum, is also higher than 2 π and can be decreased by reducing the concentration crosslinker (BMA) in the solution or by using thinner samples.

The values used in our simulation were fitted using direct methods [22]. We considered a thickness of 90 μm and the other parameters introduced were taken from the zero spatial frequency analysis *D_m_* = 1.36 μm^2^/s, polymerization constant *k_R_* = 0.014 (cm^2^/mW)1/*^γ^*, *α_T_* = 0.04 μm^−1^, attenuation of light inside the material due to the dye *α* = 0.012 μm^−1^, *M_0_* = 0.22 volume fraction, recording intensity *I_0_* = 0.5 mW/cm^2^, *n_m_* = 1.486, *n_b_* = 1.51, *n_p_* = 1.66 and *n_dark_* = 1.478.

All the simulations were done taking into account low pass filtering and introducing a real profile of the grating obtained from the experimental setup placing the CCD camera in the material plane as shown in Figure 2b.

### 4.2. Simulation of the Recording of Blazed Gratings on PVA/NaAO Photopolymer

**Figure 5:**
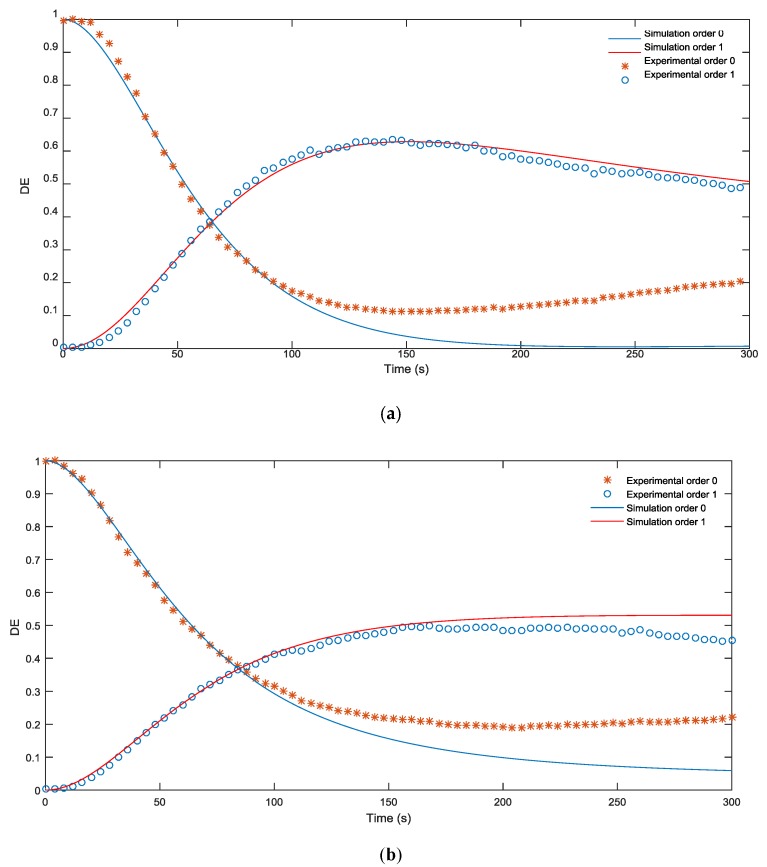
(**a**) Comparison of the simulated and experimental DE of a 672 um blazed grating recorded in PVA/NaAO material over a period of 300 s; (**b**) Comparison of the simulated and experimental DE a 336 μm blazed grating recorded in PVA/NaAO material over a period of 300 s.

To evaluate the ability of the model to simulate the behavior of different materials, we repeated the same procedure using PVA/NaAO, a variation of the photopolymer developed by our group [14], instead of the AA based photopolymer. Basically, our aim was to analyze the capability of the model to represent the recording of blazed gratings in any kind of material, regardless of its composition, and the behavior of this PVA/NaAO material in the recording of this kind of DOE. Figure 5a,b shows the DE of two blazed gratings with a period of 672 µm and 336 µm, respectively, recorded on a PVA/NaAO based photopolymer. Our model was shown to be capable of reproducing the photopolymerization that takes place during recording of a blazed grating on a material with properties different to those of the AA-PVA based material analyzed in Section 4.1. The maximum value of DE was almost 55% for an exposure time of 200 s, which is lower and slower than the 70% DE at 150 s obtained with the AA-PVA based photopolymer due to the lower values of *k_R_* and *n_p_*. This maximum value of DE may be increased using thicker samples or a higher concentration of BMA in the final solution.

In this case, we again considered a thickness of 90 μm and, as in the previous section, the other parameters introduced were taken from the zero spatial frequency analysis: *D_m_* = 1.36 μm^2^/s, polymerization constant *k_r_* = 0.010 (cm^2^/mW)1/^γ^, *α_T_* = 0.04 μm^-1^, attenuation of light inside the material *α* = 0.019 μm^−1^, *M_0_* = 0.15 volume fraction, recording intensity *I_0_* = 0.5 mW/cm^2^, *n_m_* = 1.486, *n_b_* = 1.5114, *n_p_* = 1.61 and *n_dark_* = 1.478.

**Figure 6:**
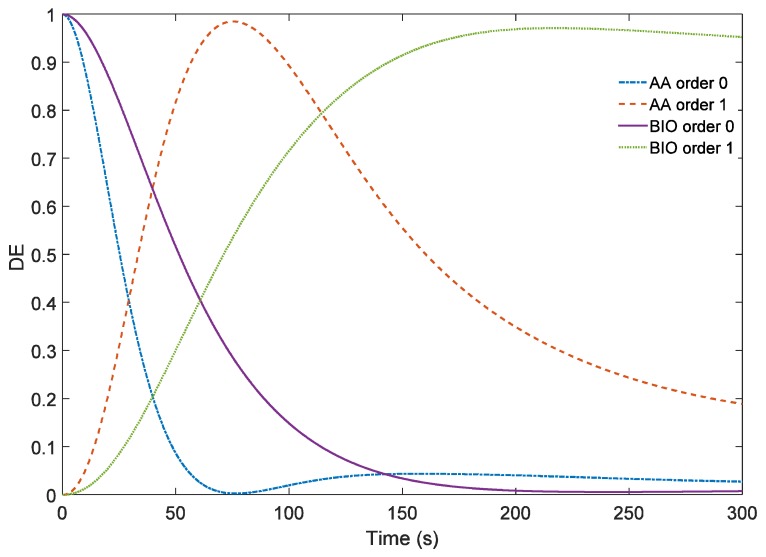
Comparison of the simulation results of AA/PVA and PVA/NaAO based materials without the addition of the low pass filtering simulation to simulate the optical system.

In Figure 6, the theoretical results without filtering are shown for both chemical compositions. Therefore, the effects of low pass filtering can be more clearly understood comparing Figure 4 and Figure 5 with Figure 6. It can be seen in the last figure a DE of 100% could be reach for the PVA/AA photopolymer and of 97% for the PVA/NaAO one. Low pass filtering led to a reduction of over 20% in the maximum DE obtained in both types of material. This maximum value was achieved faster due to the ideal recording intensity and obviously the PVA/AA photopolymer reached a DE of 100% before PVA/NaAO reached its maximum DE due to the greater difference between n_m_ and n_p_ and the higher polymerization rate. Nevertheless, in the experiments carried out, the differences between AA and NaAO materials are smaller than that expected for the ideal case, around 130 s for AA and 160 s for NaAO. 

## 5. Conclusions

In this paper, we analyzed the recording of blazed gratings in photopolymers by testing the validity of our model to simulate the recording of any kind of DOE in the desired material, regardless of its composition. Specifically, we tested our model with a PVA/AA based photopolymer and PVA/NaAO and compared the results with the experimental ones. The results showed, on the one hand, good agreement between our simulation and the experimental measurements. On the other hand, limitation of the DE maximum value was mostly due to the low pass filtering introduced by the experimental setup itself and elements such as the diaphragms disposed to eliminate pixilation before recording on the photopolymer. Without this low pass filtering, the maximum value of DE reached, 100%, was in accordance with maximum theoretically possible. We have demonstrated the usefulness of the parameters directly measured by diffractive and interference methods and, once they are introduced in the model, we reproduce the material behavior without fittings.

## Figures and Tables

**Table 1:** Composition of the liquid solution for PVA/AA photopolymer.

Component	Quantity
TEA (mL)	1.5
PVA (8% w/v) (mL)	25
AA (gr)	0.84
BMA (gr)	0.25
YE (0.8% w/v) (mL)	0.7

**Table 2:** Composition of the liquid solution for PVA/NaAO photopolymer.

Component	Quantity
TEA (mL)	1.5
PVA (8% w/v) (mL)	25
NaAO (mL)	2
BMA (gr)	0.20
YE (0.8% w/v) (mL)	0.7
